# Comparison of Anti-oncotic Effect of TRPM4 Blocking Antibody in Neuron, Astrocyte and Vascular Endothelial Cell Under Hypoxia

**DOI:** 10.3389/fcell.2020.562584

**Published:** 2020-10-19

**Authors:** Shunhui Wei, See Wee Low, Charlene Priscilla Poore, Bo Chen, Yahui Gao, Bernd Nilius, Ping Liao

**Affiliations:** ^1^Calcium Signaling Laboratory, Department of Research, National Neuroscience Institute, Singapore, Singapore; ^2^Department of Cellular and Molecular Medicine, KU Leuven, Leuven, Belgium; ^3^Duke-NUS Medical School, Singapore, Singapore; ^4^Health and Social Sciences, Singapore Institute of Technology, Singapore, Singapore

**Keywords:** ischemic stroke, TRPM4, swelling, cell death, therapeutic antibody

## Abstract

In stroke and other neurological diseases, Transient Receptor Potential Melastatin 4 (TRPM4) has been reported to cause oncotic cell death which is due to an excessive influx of sodium ions. Following stroke, hypoxia condition activates TRPM4 channel, and the sodium influx via TRPM4 is further enhanced by an increased TRPM4 expression. However, the effect of TRPM4 inhibition on oncotic cell death, particularly during the acute stage, remains largely unknown. Recently, we have developed a polyclonal antibody M4P that specifically inhibits TRPM4 channel. M4P blocks the channel via binding to a region close to the channel pore from extracellular space. Using M4P, we evaluated the acute effect of blocking TRPM4 in neurons, astrocytes, and vascular endothelial cells. In a rat stroke model, M4P co-localized with neuronal marker NeuN and endothelial marker vWF, whereas few GFAP positive astrocytes were stained by M4P in the ipsilateral hemisphere. When ATP was acutely depleted in cultured cortical neurons and microvascular endothelial cells, cell swelling was induced. Application of M4P significantly blocked TRPM4 current and attenuated oncosis. TUNEL assay, PI staining and western blot on cleaved Caspase-3 revealed that M4P could ameliorate apoptosis after 24 h hypoxia exposure. In contrast, acute ATP depletion in cultured astrocytes failed to demonstrate an increase of cell volume, and application of M4P or control IgG had no effect on cell volume change. When TRPM4 was overexpressed in astrocytes, acute ATP depletion successfully induced oncosis which could be suppressed by M4P treatment. Our results demonstrate that comparing to astrocytes, neurons, and vascular endothelial cells are more vulnerable to hypoxic injury. During the acute stage of stroke, blocking TRPM4 channel could protect neurons and vascular endothelial cells from oncotic cell death.

## Introduction

Stroke is a leading cause of disability worldwide, and of all strokes, more than 80% are ischemic (Virani et al., 2020). Cerebral edema is common among stroke patients, particularly after reperfusion. There are two types of edema, cytotoxic, and vasogenic, which are caused by cell swelling or increased vascular permeability (Rosenberg, 1999). It has been reported that stroke patients with severe edema have a mortality of up to 60–80% (Hacke et al., 1996; Berrouschot et al., 1998). Harnessing cerebral edema is thus pivotal in improving stroke outcome. However, neuroprotective agents targeting cerebral edema have not been translated into clinical treatments. For example, blockers for NMDA receptor failed to show beneficial effects due to their narrow therapeutic windows and adverse effects (Wu and Tymianski, 2018). One major reason is that the cellular and molecular mechanisms underlying cerebral edema remain largely unclear.

There are two types of cell death in stroke: cell swelling, also known as oncosis or oncotic cell death (Weerasinghe and Buja, 2012), and programmed cell death (Majno and Joris, 1995). Oncosis is caused by increased membrane permeability which leads to cell swelling. Whereas in programmed apoptosis, cell shrinkage and fragmentation into apoptotic bodies are typical features. Following oncosis or apoptosis, cells undergo irreversible necrosis and phagocytosis (Lipton, 1999). In stroke, there have a heterogeneous distribution of oncotic and apoptotic processes across affected brain regions (Charriaut-Marlangue et al., 1996). At the point of insult, the death pathway varies among cell types as well (Martin et al., 1998). Understanding the mechanisms behind such differences could pave the way for novel therapies.

Recently, novel evidence has emerged regarding the pathophysiological role of the transient receptor potential (TRP) channels in stoke (Zhang and Liao, 2015). Among the largest TRP subfamily, transient receptor potential melastatin (TRPM), several members have demonstrated as potential drug targets for stroke. Activation of TRPM2, an oxidative stress-sensitive channel, contributes to neuronal death following hypoxia/ischemia. TRPM2 inhibition has been shown to yield a neuroprotective effect in stroke reperfusion and global cerebral hypoxia (Toda et al., 2019; Hong et al., 2020). Another member TRPM7 has a unique property of both cation permeability and kinase activity (Inoue et al., 2020). TRPM7 plays an important role in cell volume regulation (Numata et al., 2007), excessive influx of the metal ions Ca^2+^, Mg^2+^, and Zn^2+^ through TRPM7 is highly toxic (Inoue et al., 2010). In addition to suppress metal ion overloading, blocking TRPM7 could also inhibit ROS production in neurons following hypoxia (Aarts et al., 2003). The most extensively studied TRPM channel in stroke is TRPM4 which has been found to play an important role in oncotic cell death (Gerzanich et al., 2009; Loh et al., 2019). TRPM4 is an ATP-sensitive non-selective cation channel that can be activated by an increased intracellular Ca^2+^ level as well as ATP depletion (Vennekens and Nilius, 2007). Both TRPM4 expression and activity are enhanced after stroke (Loh et al., 2014; Chen et al., 2019b). Under hypoxic condition, excessive Na^+^ influx via TRPM4 contributes to intracellular ionic imbalance, leading to cell swelling (Gerzanich et al., 2009; Schattling et al., 2012). Therefore, TRPM4 has become a potential target for stroke management (Walcott et al., 2012; Loh et al., 2014; Hu and Song, 2017; Chen et al., 2019b).

Sulfonylurea receptor-1 (SUR1) has been reported to interact with TRPM4, forming a SUR1-TRPM4 channel complex (Simard et al., 2010). SUR1 blocker glibenclamide is believed to block the channel complex and yields a therapeutic effect on stroke (Kurland et al., 2013). However, glibenclamide requires the presence of SUR1 which needs to be expressed at a certain level to yield a pharmacological effect (Woo et al., 2013). Without SUR1, glibenclamide could not inhibit TRPM4 channel activity (Sala-Rabanal et al., 2012). Recently, we have developed a polyclonal antibody M4P that specifically inhibits TRPM4 channel (Chen et al., 2019a). M4P binds to a region close to the channel pore from extracellular space and yields an inhibitory effect via two mechanisms: (1) blocks TRPM4 current directly; (2) down regulates TRPM4 expression on cell membrane. In a rat model of stroke reperfusion, application of M4P ameliorated reperfusion injury by protecting blood–brain barrier (BBB) and improved stroke outcome (Chen et al., 2019a).

In this study, we extended our research on M4P to its anti-oncotic effect on various cell types within the brain including neurons, astrocytes and vascular endothelial cells. Although TRPM4 has been well documented in stroke, the exact role of TRPM4 inhibition on oncotic cell death, particularly during the acute stage, remains largely unclear. Using M4P as a specific blocker for TRPM4, we are able to decipher how TRPM4 engages in disease progression immediately after ischemia which is common in stroke and many other disorders of central nervous system.

## Materials and Methods

### Animal Model of Middle Cerebral Artery Occlusion (MCAO)

This study was approved and conducted in accordance with the guidelines of the Institutional Animal Care and Use Committee of the National Neuroscience Institute, Singapore. The stroke model has been described previously (Loh et al., 2014; Chen et al., 2019a, b). In brief, male Sprague Dawley rats weighing approximately 250–280 g were anesthetized with ketamine (75 mg/kg) and xylazine (10 mg/kg) intraperitoneally. Rectal temperature was monitored using a rectal probe, connecting to the PowerLab 4/35 by a T-type pod (MLT1403&312, RET-2, AD Instruments). The left common carotid artery (CCA), internal carotid artery (ICA) and external carotid artery (ECA) were dissected out. A silicon-coated filament (0.37 mm, Cat #403756PK10, Doccol Corp., Redlands, CA, United States) was introduced into the left ICA through ECA. Cerebral blood flow of the animals was monitored by a Laser-Doppler flowmetry (moorVMS-LDF2^TM^, Moor Instruments Inc., Wilmington, DE, United States). Animals with ≤70% cerebral blood flow reduction were excluded from the study.

### Generation of Polyclonal Antibody M4P

The production of rabbit polyclonal antibody M4P has been described in our recent publication (Chen et al., 2019a). M4P was designed to bind to and block rat TRPM4 channel specifically.

### Primary Rat Cortical Neurons, Astrocytes and Rat Brain Microvascular Endothelial Cells (RBMVECs) Culture

Primary cortical neurons were prepared from embryonic day 18 (E18) pregnant Sprague Dawley rats. Fetal brain cortex was dissociated and digested for 40 min in Earle’s Balanced Salt Solution (EBSS, Thermo Fisher Scientific, Waltham, MA, United States) containing 20 U/mL papain (Worthington, Lakewood, NJ, United States). Dissociated cells were seeded on 12 mm round glass coverslips coated with poly-L-lysine and laminin and placed into 60 mm tissue culture dishes with neuron culture medium containing Neurobasal Medium, 2% B27 supplement, 1% GlutaMAX supplement (Thermo Fisher Scientific, Waltham, MA, United States). Medium was replaced 1 day after plating, and half of the medium was changed every 3 days. The cells were treated with 4 μM cytosine arabinoside from days *in vitro* (DIV) 3–6 to restrict mitotic cell proliferation and maintained for 10–21 days in neuron culture medium at 37°C.

For primary culture of cortical astrocytes, cells from cerebral cortex were digested, dissociated, and maintained for 10 days in DMEM supplemented with 10% FBS. Cultures were then treated with 10 μM Ara-C, shaken at 240 rpm for 6 h to remove oligodendrocyte precursor cells and replanted for experiments.

Rat brain microvascular endothelial cells were purchased from Cell Applications Inc (Cell Applications, San Diego, CA, United States). The culture Growth Medium and Basal medium (contains no growth supplement) were also obtained from Cell Applications Inc. Cells at passages 5–10 were used for study as per the manufacturer’s recommendation.

### Hypoxia Induction

For acute oxygen-glucose deprivation (OGD) during patch clamp recording, the cells (neurons, astrocytes, or vascular endothelial cells) were perfused with an anoxic artificial cerebrospinal fluid (aCSF) containing 5 mM NaN_3_ and 10 mM 2-deoxyglucose.

For 24-h OGD, the cells were grown in respective hypoxic media and placed in a polycarbonate hypoxia induction chamber (Modular Incubator Chamber, #27310, STEMCELL Technologies Inc., Vancouver, BC, Canada). The chamber was first flushed with a gas mixture containing 1% O_2_, 5% CO_2_, and 94% N_2_ for 5 min to purge the ambient air from the chamber. Following that, the hypoxia chamber was tightly sealed, and placed in a 37°C incubator for 24 h. The hypoxic medium for neurons contains serum-free low glucose EBSS medium, pH7.4 (1.8 mM CaCl_2_; 0.8 mM MgSO_4_; 5.3 mM KCl; 26.2 mM NaHCO_3_; 117.2 mM NaCl; 1 mM NaH_2_PO_4_; 1.85 mM D-Glucose) with 100 U/ml Penicilin-Streptomycin. For astrocytes, the hypoxic medium is DMEM with free glucose. For RBMVECs, the hypoxic medium is the Basal Medium purchased from Cell Applications (Cell Applications Inc., San Diego, CA, United States).

### Immunofluorescent Staining and Western Blot

Immunofluorescent staining was performed as previously described (Loh et al., 2014). In brief, the rats were sacrificed and perfused 1 day after stroke induction. Then, the brains were harvested and sectioned at 10 μm in thickness. Following fixation with 4% paraformaldehyde, the brain slice was incubated in 100 μl blocking serum (10% fetal bovine serum in 0.2% PBST) for 1 h. The samples were then incubated with primary antibodies overnight at 4 °C. Primary antibodies include M4P (rabbit, 10 ng/μl), anti-NeuN (MAB377, Millipore, Burlington, MA, United States, 1:250), anti-GFAP (IF03L, Millipore, Burlington, MA, United States, 1:200), and anti-vWF (AB7356, Millipore, Burlington, MA, United States, 1:200). After washing with 0.1% Triton/phosphate-buffered saline, the slides were incubated with secondary antibodies before being visualized using a laser scanning confocal microscope system (FV31S-SW Fluoview, Olympus, Tokyo, Japan). Secondary antibodies include donkey anti-rabbit conjugated with Alexa Fluor 488 and chicken anti-mouse conjugated with Alexa Fluor 594 (Catalog # A-21206, and A-21201, Life Technologies Corporation, Grand Island, NY, United States).

To perform western blot, 30 μg of total protein was resolved on 10% SDS-PAGE gels at 80V, and electrophoretically transferred to PVDF membranes (1620177, Bio-Rad, Santa Rosa, CA, United States) at 100V for 2 h at 4°C. After blocking with StartingBlock (PBS) blocking buffer (37538, Thermo Fisher Scientific, Waltham, MA, United States) for 1 h at room temperature, membranes were incubated overnight at 4°C with primary polyclonal antibody for cleaved Caspase-3 (# 9661, Cell Signaling Technology Inc., Danvers, MA, United States) and subsequent secondary goat anti-rabbit IgG (A4914, Sigma-Aldrich, St. Louis, MO, United States, 1:5000). After probing with the ECL system, the membrane was incubated with the Restore^TM^ PLUS Western Blot Stripping Buffer (#46428, Thermo Fisher Scientific, Waltham, MA, United States). Primary anti-β-actin (A1978, Sigma-Aldrich, St. Louis, MO, United States, 1:5000) and secondary goat anti-mouse IgG or (A4416 Sigma-Aldrich, St. Louis, MO, United States, 1:5000) were then used for the detection of β-actin.

### TUNEL (Terminal Deoxynucleotidyl Transferase dUTP Nick End Labeling) Assay for Apoptosis

TUNEL labeling was performed using the In Situ Cell Death Detection Kit, Fluorescein (11684795910, Roche Diagnostics, Germany). The cells cultured on glass coverslips were treated and fixed with 4% paraformaldehyde for TUNEL staining according to the manufacturer’s instructions. The cells were counterstained with DAPI to visualize the cell nuclei. The glass coverslips then mounted onto glass slides and the images were captured with a laser scanning confocal microscope system (FV31S-SW Fluoview, Olympus, Tokyo, Japan).

### PI (Propidium Iodide) Assay

The treated cells on glass coverslips were added with culture media containing 1:100 dilution of 1 mg/mL of the PI stock solution (P1304MP, Thermo Fisher Scientific, Waltham, MA, United States). The cells were incubated for 15 min in a cell culture humidified incubator set at 37°C with 5% CO_2_. After which, the cells were washed three times with PBS and fixed with 4% paraformaldehyde. The cells were counterstained with DAPI and mounted onto glass slides. The images were captured with the confocal microscope.

### Transfection

Transfection was performed on cell cultures on coated coverslips in 24-well plates using Lipofectamine-2000 (Life Technologies, Carlsbad, CA, United States) according to the manufacturer’s instructions. The transfection mix consisted 1 μg plasmid DNA and 1.5 μl Lipofectamine^TM^ 2000 reagent for each well. Mouse TRPM4 (pIRES-EGFP-TRPM4) was transiently expressed in astrocytes, TRPM2 or TRPM7 was co-transfected with GFP-containing vector pEGFP-N1 in HEK 293 cells. Mouse TRPM2 (Myc-DDK-tagged) was purchased from OriGene (CAT # MR225380, OriGene, Technologies Inc., Rockville, MD, United States), pcDNA4/TO mTRPM7 was a gift from Andrew Scharenberg (Addgene plasmid # 45482; RRID: Addgene_45482)^1^.

### Electrophysiology

Whole-cell patch clamp was used to examine the electrophysiological properties of the cultured cells at room temperature. Patch electrodes were pulled using a Flaming/Brown micropipette puller (P-1000, Sutter Instrument, Novato, CA, United States) and polished with a microforge (MF-200, WPI Inc., Sarasota, FL, United States). Whole-cell currents were recorded using a patch clamp amplifier (Multiclamp 700B equipped with Digidata 1440A, Molecular Devices, San Jose, CA, United States). The bath solution contained (in millimole/liter): NaCl 140, CaCl_2_ 2, KCl 2, MgCl_2_ 1, glucose 20, and HEPES 20 at pH 7.4. The internal solution contained (in millimole/liter): CsCl 156, MgCl_2_ 1, EGTA 10, and HEPES 10 at pH 7.2 adjusted with CsOH (Schattling et al., 2012). Rabbit IgG or M4P was added into bath solution at a concentration of 20.8 μg/ml for 30 min before recording. Ischemia/Hypoxia was induced by applying a bath solution containing 5 mM NaN_3_ and 10 mM 2-deoxyglucose (2-DG) continuously through a MicroFil (34 Gauge, WPI Inc., United States) around 10 μm away from the recording cells. The flow rate was 200 μl/min. The current–voltage relations were measured by applying voltage ramps for 250 ms from –100 to +100 mV at a holding potential of −70 mV for neurons, or 0 mV for astrocyte and vascular endothelial cells. The sampling rate was 20 kHz and the filter setting was 1 KHz. Data were analyzed using pClamp 10, version 10.2 (Molecular Devices, San Jose, CA, United States). Cell membrane capacitance was recorded by online capacitance measurement in Clampex of pClamp 10 with the update rate at 100 Hz and Membrane Test function was used to set the compensation every 1 min). The images of every cell were also captured before and after hypoxia induction to compare the changes of cell size.

For TRPM2 current recording in HEK 293 cells, the bath solution contained (in millimole/liter): NaCl 140, CaCl_2_ 2, KCl 2, MgCl_2_ 1, glucose 20 and HEPES 20 at pH 7.4. The pipette solution contained (in millimole/liter): Cs-methanesulfonate 145, NaCl 8, EGTA 1 and HEPES 10 at pH 7.2 adjusted with CsOH (Du et al., 2009), free internal Ca^2+^ [Ca^2+^]_*i*_ was adjusted to 100 μM based on calculations using Maxchelator^2^ (Du et al., 2009). For TRPM7 current recording in HEK 293 cells, 24 h after transfection, TRPM7 expression was induced by adding 1 μg/ml tetracycline to DMEM culture medium. Whole-cell patch clamp was performed 18–26 h after induction, the divalent-free bath solution was used which containing (in millimole/liter : NaCl 145, EGTA 10, glucose 10 and HEPES 20 at pH 7.4 (Jiang et al., 2005), the pipette solution contained (in millimole/liter): Cs-methanesulfonate 145, NaCl 8, EGTA 1 and HEPES 10 at pH 7.2 adjusted with CsOH. For all HEK 293 cells recording, 250-ms voltage ramps from −100 to +100 mV was applied. Holding potential: 0 mV.

### Statistical Analysis

Data are expressed as the mean ±S.E.M. Statistical analyses were performed using GraphPad Prism version 6.0. Two-tailed unpaired student’s *t-*test was used to compare two means. One-way ANOVA with Bonferroni’s multiple comparison test was used to compare ≥3 means.

## Results

### M4P Detects Upregulated TRPM4 Expression in Ischemic Stroke

**FIGURE 1:**
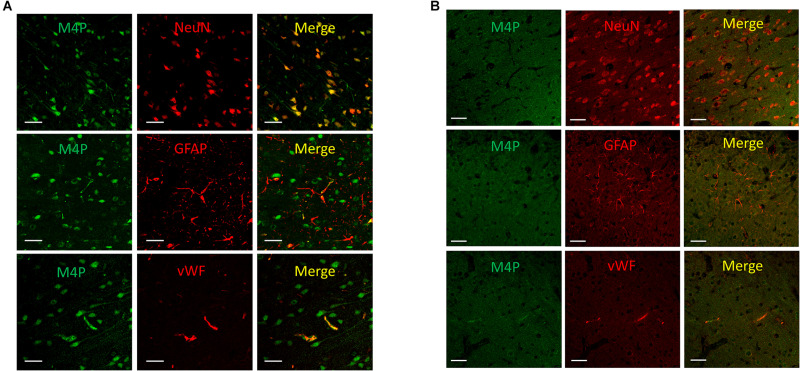
Using M4P to detect TRPM4 expression in a rat model of stroke. **(A)** Brain slices from ipsilateral hemisphere were double stained with M4P and neuronal marker NeuN (upper panel), astrocyte marker GFAP (middle panel), or endothelial cell marker vWF (lower panel). **(B)** Immunofluorescent staining of TRPM4 expression in the contralateral hemisphere. Scale bars 50 μm.

To study the expression of TRPM4 in various cell types after stroke, a permanent MCAO model was created in male Sprague Dawley rats. It has been reported that the expression of TRPM4 is low in all cell types in the contralateral hemisphere after stroke (Loh et al., 2014). In this study, TRPM4 expression in the ipsilateral hemisphere was detected by the rat TRPM4 specific antibody M4P. We focus on the area close to the infarct core where the tissue is still viable, but has been affected by hypoxia. M4P was found to co-localize with neuronal marker NeuN and endothelial cell marker vWF. In contrast, few GFAP positive astrocytes were stained by M4P (Figure 1A). In contralateral hemisphere, the expression of TRPM4 was low in all cell types (Figure 1B). This result suggests that comparing to astrocytes, TRPM4 expression is higher in neuron and vascular endothelial cell following stroke induction.

### M4P Inhibits TRPM4 Activity in Cortical Neurons and Ameliorate Hypoxia-Induced Cell Swelling

**FIGURE 2:**
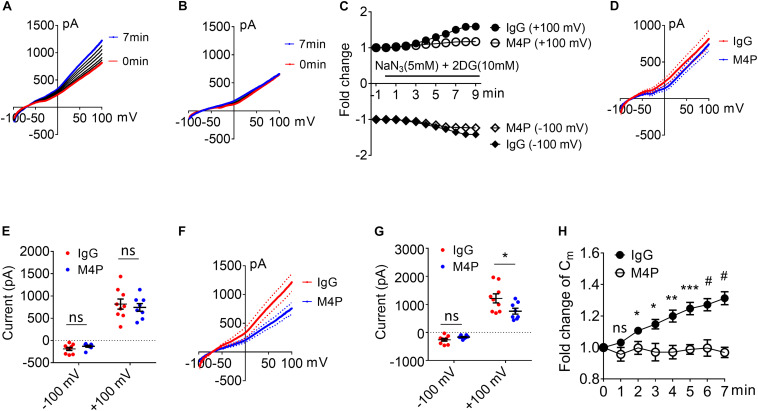
M4P blocks TRPM4 activity in cultured cortical neurons and prevents hypoxia-induced cell swelling. **(A)** Sample traces from a cortical neuron pre-treated with control rabbit IgG (20.8 μg/ml) for 30 min. Acute hypoxia was induced by adding 5 mM NaN_3_ and 10 mM 2-DG to the neuron for 7 min. 250-ms voltage ramps from –100 to +100 mV was applied at 1 min interval. Holding potential: –70 mV. The pipette solution contained a calculated 7.4 μM free Ca^2+^. **(B)** Sample traces from a cortical neuron pre-treated with M4P at 20.8 μg/ml for 30 min. The recording protocol is similar to that described in **(A)**. **(C)** Time-dependent current changes at ±100 mV from **(A,B)**. All currents were normalized to baseline before hypoxic induction. **(D)** Summarized current-voltage relationship at 0 min under hypoxia for control IgG (*n* = 9) and M4P (*n* = 8) treatments. **(E)** Summary of currents at -100 and 100 mV at 0 min under hypoxia. Control IgG: *n* = 9; M4P: *n* = 8. **(F)** Summarized current-voltage relationship at 7 min under hypoxia for control IgG (*n* = 9) and M4P (*n* = 8) treatments. **(G)** Summary of currents at -100 and 100 mV at 7 min under hypoxia. Control IgG: *n* = 9; M4P: *n* = 8. **(H)** Time course of normalized membrane capacitance (C_*m*_) under hypoxic conditions. Control IgG: *n* = 9; M4P: *n* = 8. Statistical analysis was performed by two tailed unpaired student’s *t-*test for **(E,G)**, and two-way ANOVA test with *post hoc* Bonferroni’s analysis for H. *p < 0.05, **p < 0.01, ***p < 0.001, and #*p* < 0.0001, ns, non-significant.

To investigate the role of TRPM4 inhibition on neurons, cortical neurons were pre-incubated with M4P or control rabbit IgG for 30 min before patch clamping. To induce acute hypoxia, 5 mM sodium azide (NaN_3_) and 10 mM 2-deoxyglucose (2-DG) was applied to the cells during recording to inhibit mitochondrial respiration and energy metabolism (Chen et al., 2019a). Current-voltage relationship of cortical neurons were obtained by whole-cell patch clamp ramp protocols applied from -100 to +100 mV. The neurons were held at −70 mV to reflect the native resting membrane potential. Under control IgG treatment, the current exhibited a continuous rise during the 7-min hypoxia incubation (Figures 2A,C). Whereas no current increase was observed with M4P treatment (Figures 2B,C). Before hypoxia induction, we did not observe difference between M4P and IgG treated cells (Figures 2D,E). We further compared the currents at 7 min after hypoxia induction, and found that the TRPM4 activity was significantly blocked by M4P application. At +100 mV, the current was reduced by 37.5%, from 1214.3 ± 160.9 pA in control IgG group to 758.3 ± 97.8 pA in M4P group (Figures 2F,G). This result suggests that the current increase by acute hypoxia is mainly contributed by TRPM4 channel. Next, we measured cell volume change after acute hypoxia. As cell membrane capacitance (C_*m*_) has been reported to have a positive linear correlation with cell volume (Satoh et al., 1996), we monitored C_*m*_ changes as a surrogate of cell volume during the 7-min hypoxia treatment (Figure 2H). In control IgG group, the C_*m*_ was found to increase gradually. At 7 min, the C_*m*_ has reached 131.3% of baseline level before hypoxia induction. In M4P treated cells, no change was observed during the 7-min hypoxia treatment.

**FIGURE 3:**
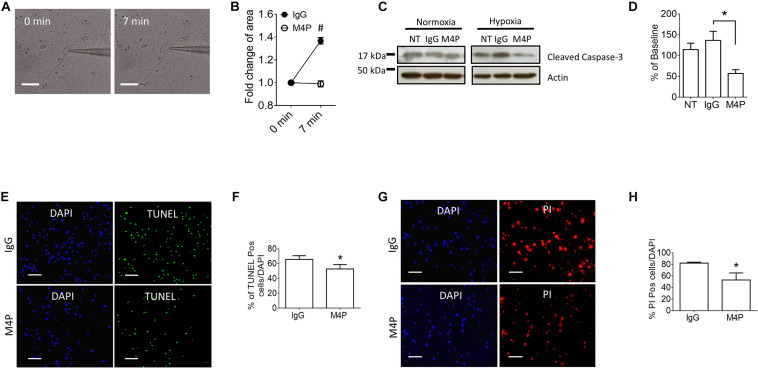
M4P inhibits hypoxia-induced cell swelling and cell death in cortical neurons. **(A)** Representative images of a neuron taken before (0 min) and after hypoxia induction (7 min). Scale bars: 20 μm. **(B)** Summary of normalized cell body areas with IgG (*n* = 9) or M4P (*n* = 8) treatment. **(C)** Western blot of cleaved Caspase-3 in cortical neurons incubated with IgG or M4P at 10.4 μg/ml for 24 h, NT, non-treatment. **(D)** Summary of Cleaved Caspase-3 expression (normalized to actin) under hypoxia. *n* = 4 for IgG, M4P, and NT. **(E)** TUNEL assay of cortical neurons under hypoxia condition for 24 h treated with IgG (upper panel) or M4P (lower panel). Scale bars: 50 μm. **(F)** Quantification of TUNEL positive cells (TUNEL Pos cells), *n* = 4. **(G)** PI assay of cortical neurons under hypoxia condition for 24 h treated with IgG (upper panel) or M4P (lower panel). Scale bar: 50 μm. **(H)** Quantification of PI positive cells (PI Pos cells), *n* = 3. In **(B,F,H)**, statistical analysis was performed by two tailed unpaired student’s *t-*test; in **(D)**, by one-way ANOVA with Bonferroni’s *post hoc* analysis. *p < 0.05 and #*p* < 0.0001.

To validate the results from C_*m*_ measurement, we further captured the images of the cells before and after hypoxia treatment, and analyzed cell area using ImageJ software. Sample images taken at 0 min and 7 min of the same cell clearly showed that acute hypoxia treatment could induce cell area increase (Figure 3A). Summary of cell area quantification demonstrated that in IgG treatment group, the cell area at 7 min was increased to 136.8 ± 2.9% of baseline (Figure 3B), similar to the result from C_*m*_ measurement. Again, neurons with M4P treatment showed no significant change in cell area after 7-min hypoxia induction (Figure 3B). This result clearly indicates that acute hypoxia is able to induce cell swelling in cortical neurons, and blocking TRPM4 current with M4P antibody could attenuate cytotoxic edema. To investigate whether M4P application could protect neurons from apoptotic cell death, we cultured the cells in a hypoxic medium and incubated them in a hypoxia/anoxia chamber containing 1% O_2_, 5% CO_2_, and 94% N_2_ for 24 h. Cleaved Caspase-3, a marker of apoptosis, was detected with western blot (Figure 3C). Analysis of cleaved Caspase-3 expression revealed that M4P treatment successfully attenuated hypoxia-induced apoptosis (Figure 3D). TUNEL assay (Figures 3E,F), PI staining (Figures 3G,H) also revealed that M4P could ameliorate apoptosis after 24 h hypoxia exposure.

### M4P Inhibits TRPM4 Activity in RBMVEC and Ameliorate Hypoxia-Induced Cell Swelling

**FIGURE 4:**
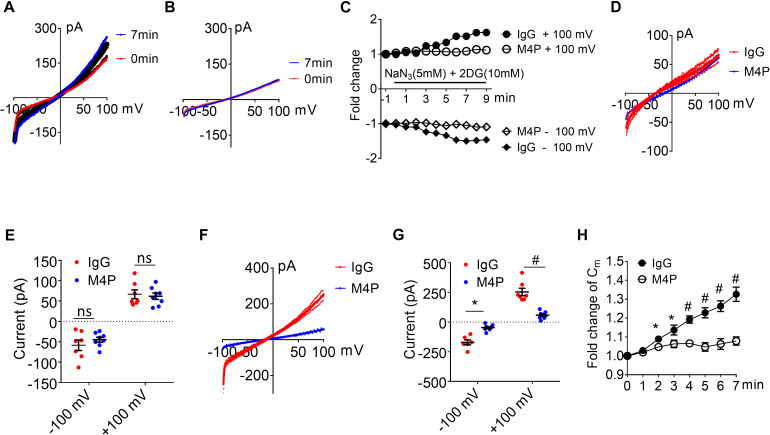
M4P blocks TRPM4 activity in cultured rat brain microvascular endothelial cells (RBMVECs) and prevents hypoxia-induced cell swelling. **(A)** Sample traces from an RBMVEC cell pre-treated with control rabbit IgG (20.8 μg/ml) for 30 min. Acute hypoxia was induced by adding 5 mM NaN_3_ and 10 mM 2-DG to the cell for 7 min. 250-ms voltage ramps from -100 to +100 mV was applied at 1 min interval. Holding potential: 0 mV. The pipette solution contained a calculated 7.4 μM free Ca^2+^. **(B)** Sample traces from an RBMVEC cell pre-treated with M4P at 20.8 μg/ml for 30 min. The recording protocol is similar to that described in **(A)**. **(C)** Time-dependent current changes at ±100 mV from **(A,B)**. All currents were normalized to baseline before hypoxic induction. **(D)** Summarized current-voltage relationship at 0 min under hypoxia for control IgG (*n* = 7) and M4P (*n* = 8) treatments. **(E)** Summary of currents at -100 and 100 mV at 0 min under hypoxia. Control IgG: *n* = 7; M4P: *n* = 8. **(F)** Summarized current-voltage relationship at 7 min under hypoxia for control IgG (*n* = 7) and M4P (*n* = 8) treatments. **(G)** Summary of currents at -100 and 100 mV at 7 min under hypoxia. Control IgG: *n* = 7; M4P: *n* = 8. **(H)** Time course of normalized membrane capacitance (C_*m*_) under hypoxic conditions. Control IgG: *n* = 7; M4P: *n* = 8. Statistical analysis was performed by two tailed unpaired student’s *t-*test for **(E,G)**, and two-way ANOVA test with *post hoc* Bonferroni’s analysis for **(H)**. *p < 0.05, #*p* < 0.0001, ns, non-significant.

The integrity of the BBB is compromised in stroke, and cerebral vascular endothelial cells are critical to sense hypoxia and respond by disrupting barrier function (Shah and Abbruscato, 2014). To study the role of TRPM4 in vascular endothelial cells, we cultured rat brain microvascular endothelial cells (RBMVECs). Current-voltage relationship of RBMVECs were first obtained by whole-cell patch clamp using ramp protocols applied from -100 to +100 mV. The potential was held at 0 mV which is different to the −70 mV for neurons. Similarly as to neurons, RBMVECs were incubated with 20.8 μg/ml IgG or M4P for 30 min before patch clamping. Under control IgG treatment, the current was gradually rising during the 7-min hypoxia incubation (Figures 4A,C). Whereas no current increase was observed with M4P treatment (Figures 4B,C). Similar as in neurons, we did not observe an effect of M4P on vascular endothelial cells before hypoxia treatment (Figures 4D,E). We further compared the currents at 7 min after hypoxia induction, and found that the TRPM4 activity was significantly blocked by M4P application. At +100 mV, the current was reduced by 77%, from 254.2 ± 29.0 pA in control IgG group to 58.4 ± 11.0 pA in M4P group (Figures 4F,G). This 77% reduction is even higher than the 37.5% reduction found in neurons. At −100 mV, the current from RBMVECs was reduced by 81%, from −248.4 ± 74.0 pA in control IgG group to −46.16 ± 9.0 pA in M4P group (Figures 4F,G). This result suggests that comparing to neurons, acute hypoxia is likely to induce relatively higher current increase in vascular endothelial cells which can be inhibited by blocking TRPM4 channel. Next, we examined whether acute hypoxia could change cell volume in RBMVECs. The membrane capacitance (C_*m*_) was measured at different time points under hypoxic conditions (Figure 4H). In control IgG treated cells, the C_*m*_ started to increase significantly as early as 2 min after hypoxia induction. By 7 min, the C_*m*_ was increased to 132 % of baseline at 0 min. In contrast, M4P treatment successfully inhibited C_*m*_ increase induced by hypoxia (Figure 4H).

**FIGURE 5:**
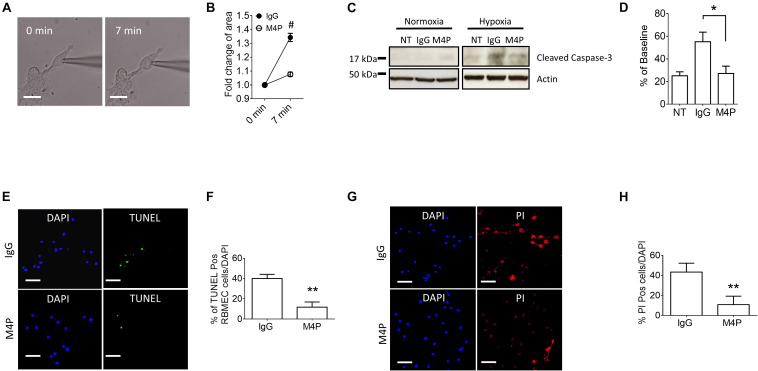
M4P inhibits hypoxia-induced cell swelling and cell death in RBMVECs. **(A)** Representative images of an RBMVEC cell taken before (0 min) and after hypoxia induction (7 min). Scale bars: 20 μm. **(B)** Summary of normalized cell body areas with IgG (*n* = 10) or M4P (*n* = 10) treatment. **(C)** Western blot of cleaved Caspase-3 in RBMVECs incubated with IgG or M4P at 10.4 μg/ml for 24 h. NT: non-treatment. **(D)** Summary of Cleaved Caspase-3 expression (normalized to actin) under hypoxia. *n* = 4 for IgG, M4P, and NT. **(E)** TUNEL assay of RBMVECs under hypoxia condition for 24 h. Treatment of IgG: upper panel, M4P: lower panel. Scale bars: 50 μm. **(F)** Quantification of TUNEL positive cells (TUNEL Pos cells), *n* = 5. **(G)** PI assay of RBMVECs under hypoxia treated with IgG (upper panel) or M4P (lower panel). Scale bar: 50 μm. **(H)** Quantification of PI positive cells (PI Pos cells), *n* = 4. In **(B,F,H)**, statistical analysis was performed by two tailed unpaired student’s *t-*test; in **(D)**, by one-way ANOVA with Bonferroni’s *post hoc* analysis. *p < 0.05, **p < 0.01, and #*p* < 0.0001.

We further measured cell area changes in RBMVECs before and after hypoxia treatment by image analysis. Sample images taken at 0 and 7 min of the same cell clearly showed that acute hypoxia treatment could induce cell area increase (Figure 5A). At 7 min, the cell area was increased to 134.3 ± 3.0 % of baseline in control IgG group (Figure 5B), similar to the result from C_*m*_ measurement in Figure 4H. Again, M4P application significantly reduced the change of cell area under hypoxic conditions (Figure 5B). After 24-hr incubation under OGD, western blot on cleaved Caspase-3 showed that M4P treatment could reduce hypoxia-induced apoptosis (Figures 5C,D). TUNEL assay (Figures 5E,F), PI staining (Figures 5G,H) also revealed that M4P could ameliorate hypoxia-induced apoptosis after 24 h hypoxia exposure. This result clearly indicates that acute hypoxia is able to induce cell swelling in vascular endothelial cells, and blocking TRPM4 current with M4P antibody could attenuate cytotoxic edema and apoptosis.

### M4P Doesn’t Affect Astrocytes During Acute Hypoxia

**FIGURE 6:**
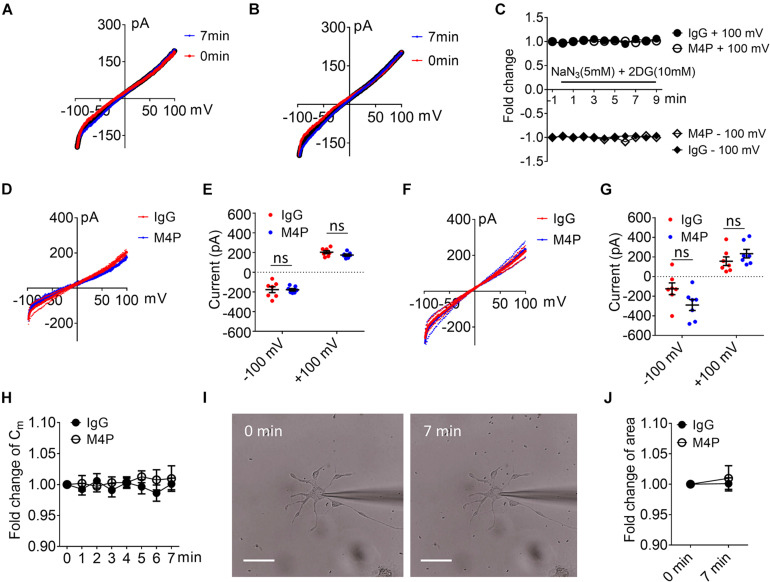
Effect of M4P on cultured rat astrocytes. **(A)** Sample traces from an astrocyte pre-treated with control rabbit IgG (20.8 μg/ml) for 30 min. Acute hypoxia was induced by adding 5 mM NaN_3_ and 10 mM 2-DG to the cell for 7 min. 250-ms voltage ramps from -100 to +100 mV was applied at 1 min interval. Holding potential: 0 mV. The pipette solution contained a calculated 7.4 μM free Ca^2+^. **(B)** Sample traces from an astrocyte pre-treated with M4P at 20.8 μg/ml for 30 min. The recording protocol is similar to that described in **(A)**. **(C)** Time-dependent current changes at ±100 mV from **(A,B)**. All currents were normalized to baseline before hypoxic induction. **(D)** Summarized current-voltage relationship at 0 min under hypoxia for control IgG (*n* = 7) and M4P (*n* = 7) treatments. **(E)** Summary of currents at -100 and 100 mV at 0 min under hypoxia. Control IgG: *n* = 7; M4P: *n* = 7. **(F)** Summarized current-voltage relationship at 7 min under hypoxia for control IgG (*n* = 7) and M4P (*n* = 7) treatments. **(G)** Summary of currents at -100 and 100 mV at 7 min under hypoxia. Control IgG: *n* = 7; M4P: *n* = 7. **(H)** Time course of normalized membrane capacitance (C_*m*_) under hypoxic conditions. Control IgG: *n* = 7; M4P: *n* = 7. **(I)** Representative images of an astrocyte taken before (0 min) and after hypoxia induction 7 min. Scale bars: 20 μm. **(J)** Summary of normalized cell body areas with IgG (*n* = 7) or M4P (*n* = 7) treatment. In **(E,G,J)**, statistical analysis was performed by two tailed unpaired student’s *t-*test, in **(H)**, by two-way ANOVA test with *post hoc* Bonferroni’s analysis. No significant difference was observed in all tests, ns, non-significant.

To examine the effect of blocking TRPM4 in astrocytes, we first measured the current-voltage relationship in primary cultured astrocytes under 7-min hypoxic conditions (Figures 6A–C). The cells were pre-incubated with control IgG and M4P for 30 min, and the 250-ms voltage ramps were applied at 1 min interval. Acute hypoxia induction did not elicit current change in both IgG and M4P treated astrocytes. We did not observe an effect of M4P on astrocytes before hypoxia treatment (Figures 6D,E). Comparing the currents after 7 min hypoxia incubation, there was no difference between IgG and M4P treated cells (Figures 6F,G), *p* = 0.07 and *p* = 0.24 for −100 mV and +100 mV, respectively. Time course of normalized membrane capacitance (C_*m*_) did not show difference between IgG (*n* = 7) or M4P (*n* = 7) groups (Figure 6H). Likewise, images taken before and after hypoxia induction revealed no changes in terms of cell areas (Figures 6I,J).

**FIGURE 7:**
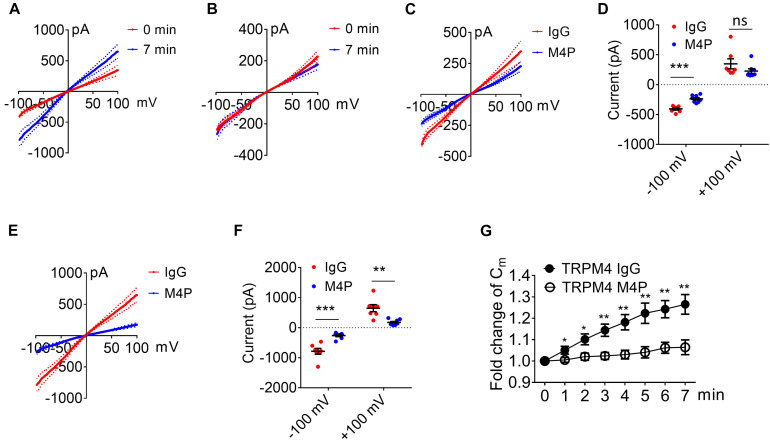
TRPM4 overexpression induces oncosis under hypoxia in cultured rat astrocytes. **(A)** Summarized current-voltage relationship of TRPM4 overexpressed astrocytes (*n* = 7) at 0 min and 7 min under hypoxia. There cells were pre-treated with control rabbit IgG (20.8 μg/ml) for 30 min. Acute hypoxia was induced by adding 5 mM NaN_3_ and 10 mM 2-DG to the cell for 7 min. 250-ms voltage ramps from -100 to +100 mV was applied at 1 min interval. Holding potential: 0 mV. The pipette solution contained a calculated 7.4 μM free Ca^2+^. **(B)** Summarized current-voltage relationship at 0 min and 7 min under hypoxia for TRPM4 overexpressed astrocytes (*n* = 7) pre-treated with M4P (20.8 μg/ml) for 30 min. **(C)** Summarized current-voltage relationship at 0 min under hypoxia for control IgG (*n* = 7) and M4P (*n* = 7) treatments. **(D)** Summary of currents at -100 and 100 mV at 0 min under hypoxia. Control IgG: *n* = 7; M4P: *n* = 7. **(E)** Summarized current-voltage relationship at 7 min under hypoxia for control IgG (*n* = 7) and M4P (*n* = 7) treatments. **(F)** Summary of currents at -100 and 100 mV at 7 min under hypoxia. Control IgG: *n* = 7; M4P: *n* = 7. **(G)** Time course of normalized membrane capacitance (C_*m*_) under hypoxic conditions. Control IgG: *n* = 7; M4P: *n* = 7. In **(D,F)**, statistical analysis was performed by two tailed unpaired student’s *t-*test, in **(G)**, by two-way ANOVA test with *post hoc* Bonferroni’s analysis. *p < 0.05, **p < 0.01, ***p < 0.001, ns, non-significant.

Next, we examined whether the lack of response to hypoxia is because the expression level of TRPM4 is low in astrocytes. We overexpressed mouse TRPM4 in astrocytes and treated with IgG and M4P under hypoxic condition. The results from TRPM4 overexpressed astrocytes showed that TRPM4 current was increased under hypoxia with IgG treatment (Figure 7A). In contrast, M4P treatment successfully inhibited TRPM4 currents (Figure 7B). M4P reduced TRPM4 current significantly at –100 mV before hypoxia induction (−405.90 ± 19.37 pA for IgG treatment, −236.78 ± 21.05 pA for M4P treatment) (Figures 7C,D). The effect of TRPM4 inhibition was more remarkable at 7 min after hypoxia treatment (at −100 mV, −787.84 ± 98.32 pA for IgG treatment, and −264.45 ± 33.10 pA for M4P treatment; at 100 mV, 648.46 ± 117.41 pA for IgG treatment, 176.81 ± 32.37 pA for M4P treatment) (Figures 7E,F). Importantly, in TRPM4 overexpression astrocytes, acute hypoxia successfully induced cell swelling in IgG group, and M4P treatment again inhibited cell volume increase (Figure 7G).

### M4P Does Not Affect TRPM2 and TRPM7 Channels

**FIGURE 8:**
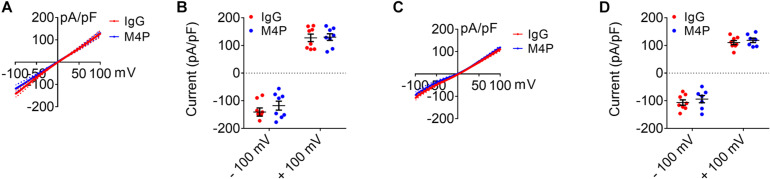
Effect of M4P on TRPM2 and TRPM7 overexpressed HEK 293 cells. **(A)** Summarized current-voltage relationship for TRPM2 overexpressed HEK 293 cells pre-treated with control rabbit IgG (20.8 μg/ml) (*n* = 8) or M4P (20.8 μg/ml) (*n* = 8) for 30 min. 250-ms voltage ramps from -100 to +100 mV was applied. Holding potential: 0 mV. The pipette solution contained a calculated 100 μM free Ca^2+^. **(B)** Summary of current density for TRPM2 overexpressed HEK 293 cells at -100 and 100 mV. Control IgG: *n* = 8; M4P: *n* = 8. **(C)** Summarized current-voltage relationship for TRPM7 overexpressed HEK 293 cells pre-treated with control rabbit IgG (20.8 μg/ml) (*n* = 8) or M4P (20.8 μg/ml) (*n* = 7) for 30 min. 250-ms voltage ramps from -100 to +100 mV was applied. Holding potential: 0 mV. Divalent-free bath solution was used. **(D)** Summary of current density for TRPM7 overexpressed HEK 293 cells at -100 and 100 mV. Control IgG: *n* = 8; M4P: *n* = 7. In **(B,D)**, statistical analysis was performed by two tailed unpaired student’s *t*-test, no significant difference was observed between IgG and M4P treatment in all tests.

We have shown previously that M4P does not interact with TRPM5, a close member of TRPM4 (Chen et al., 2019a). Here, we further examined the effect of M4P on TRPM2 and TRPM7 which have been reported to play a role in hypoxia (Aarts et al., 2003; Toda et al., 2019; Hong et al., 2020). In TRPM2 overexpressed HEK 293 cells, no difference was identified for TRPM2 currents activated by 100 μM [Ca^2+^]_*i*_ between IgG and M4P treatments (Figures 8A,B). This result suggests that M4P doesn’t affect TRPM2 channel. In TRPM7 overexpressed HEK 293 cells, to amplified TRPM7 channel current, divalent-free bath solution was used with the removal of both Ca^2+^ and Mg^2+^. No difference was identified for TRPM7 currents between IgG and M4P treatments (Figures 8C,D), suggesting that M4P doesn’t interact with TRPM7 channel.

## Discussion

It is known that cerebral edema evolves in stages, and each stage is characterized by distinct morphological and molecular changes. Cytotoxic edema, or cellular swelling, appears within minutes after acute central nervous system injuries such as stroke (Stokum et al., 2016). Therefore, targeting cellular swelling within the hyperacute stage following disease onset has a great therapeutic potential. In this study, we found that cultured neurons and vascular endothelial cells exhibited cell swelling immediately after hypoxic induction. On the contrary, acute hypoxia failed to induce cell swelling in astrocytes, suggesting that the pathophysiological process varies among different cells in the brain after hypoxia. This finding corroborates earlier reports showing that comparing to neurons, astrocytes are more resistant to OGD (Almeida et al., 2002; Panickar and Norenberg, 2005).

To unravel the underlying mechanism of cell swelling, we first examined the expression of TRPM4 channel in a rat model of stroke. It has been reported that under ischemic/hypoxic conditions, influx of monovalent cations via TRPM4 causes cell swelling and necrotic cell death (Simard et al., 2006, 2012; Gerzanich et al., 2009). Within the area surrounding the infarct core, TRPM4 expression demonstrated a differential profile depending on cell types. Both neurons and vascular endothelial cells were positively stained by M4P, whereas astrocytes showed negative staining. This result is in line with our earlier reports showing that TRPM4 is upregulated in neurons and vascular endothelial cells following stroke induction (Loh et al., 2014; Chen et al., 2019a, b). It should be noted that in these reports, TRPM4 level is very low in healthy tissues, and its upregulation occurs at least 2–6 h after stroke induction (Loh et al., 2014; Chen et al., 2019b). So, it is critical to understand whether TRPM4, at its baseline level, could affect cell functions immediately after hypoxic induction, particularly on cell volume changes.

In cultured neurons and vascular endothelial cells, acute hypoxia enhances membrane permeability which can be inhibited by TRPM4 blocking antibody M4P, suggesting that TRPM4 activation is a major cause of ionic influx at this stage. The corresponding sodium influx via TRPM4 will cause an osmotic imbalance, leading to water entry and subsequent cell swelling or cytotoxic edema (Loh et al., 2019). As expected, we observed a significant increase of cell volume as early as 2 min after hypoxia induction in control IgG treated cells. Given that the cell swelling was almost completely abrogated by M4P treatment, we believe that TRPM4 is a major and important contributor to oncosis in neurons and vascular endothelial cells immediately after hypoxia occurs. As the protein level of TRPM4 only increases several hrs after hypoxia (Loh et al., 2014; Chen et al., 2019b), the enhanced channel activity is most likely caused by the intracellular hypoxic changes which are characterized by a decrease of ATP level and an increase of Ca^2+^ (Vennekens and Nilius, 2007). Our results from neurons prove that M4P only suppresses current increase following hypoxia induction, but has no effect on the baseline currents under normoxia condition. A similar result was observed in vascular endothelial cells. Therefore, blocking TRPM4 can yield a protective role immediately after hypoxia attack, even before the upregulation of TRPM4 protein.

In contrast to neuron and vascular endothelial cell, acute hypoxia induction had no effect on current flow and cell volume in astrocytes, indicating that astrocytes are more resistant to hypoxia. Furthermore, neither M4P nor control IgG treatment could affect currents, suggesting that TRPM4 channel does not play a role in astrocyte during acute hypoxia. Importantly, when TRPM4 was transfected into astrocytes, acute hypoxia successfully induced cell swelling which could be inhibited by M4P treatment. These data suggest that with the absence of TRPM4, astrocytes are more resistance to acute hypoxia-induced oncosis. Our results do not contradict with a previous study showing that TRPM4 contribute to cell swelling in astrocytes (Stokum et al., 2018). In that study, astrocytes were activated overnight with TNFα, IFNγ, and LPS, a strong stimulus to initiate gliosis. TRPM4 expression is thereby upregulated and likely to cause oncosis. Whereas in our study, astrocytes were recorded without pre-stimulation. Thus, one can hypothesize that immediately after hypoxia, the baseline level of TRPM4 in astrocytes is too low to yield any impact on cell volume change, if there is any.

In healthy brain, large molecules such as antibody are unable to cross BBB. However, under pathological conditions such as stroke and head injury, impaired BBB could no longer serve as an effective barrier to therapeutic antibodies (Yu et al., 2013). In head injury, BBB is disrupted immediately, and therapeutic antibodies can easily enter the brain almost immediately. Whereas in stroke, BBB integrity is relatively intact right after occlusion. Hence, M4P application during this stage could mainly act on vascular endothelial cells and prevent acute oncotic cell death. This is of clinical importance as preserved BBB will attenuate vasogenic edema and improve the efficacy of reperfusion therapy by ameliorating associated side effects. Recently, we have observed this protective effect of M4P in a rat stroke model (Chen et al., 2019a). When BBB becomes leaky at later point of time, M4P can easily enter the brain and bind to neurons. Therefore, blocking TRPM4 for a long time could still yield a beneficial effect as manifested by our results showing a reduction of apoptosis in both neurons and vascular endothelial cells.

## Conclusion

In conclusion, our data have uncovered a previously unknown function of TRPM4 on cell swelling during acute ischemia/hypoxia. During the hyperacute stage of stroke or other acute brain injuries, neurons and vascular endothelial cells, rather than astrocytes, are more likely to develop cell swelling or cytotoxic edema. TRPM4 plays a key role during such process, and blocking TRPM4 is a good therapeutic approach to prevent oncosis.

## Data Availability Statement

The raw data supporting the conclusion of this article will be made available by the authors, without undue reservation.

## Ethics Statement

This study was approved and conducted in accordance with the guidelines of the Institutional Animal Care and Use Committee of the National Neuroscience Institute, Singapore.

## Author Contributions

PL and BN conceived and directed the project. SW and PL conceived, analyzed the data, and wrote the manuscript. SW, BC, YG, SL, and CP performed the experiments and analyzed the data. All authors contributed to the article and approved the submitted version.

## Conflict of Interest

The authors declare that the research was conducted in the absence of any commercial or financial relationships that could be construed as a potential conflict of interest.

## Footnotes

1 https://n2t.net/addgene:45482

2 https://somapp.ucdmc.ucdavis.edu/pharmacology/bers/maxchelator/webmaxc/webmaxcS.htm
